# Violated expectations during locomotion through virtual environments: Age effects on gaze guidance

**DOI:** 10.1167/jov.25.13.11

**Published:** 2025-11-20

**Authors:** Sophie Meissner, Jochen Miksch, Lena Würbach, Sascha Feder, Sabine Grimm, Wolfgang Einhäuser, Jutta Billino

**Affiliations:** 1 Experimental Psychology, Justus Liebig University Giessen, Germany; 2 Center for Mind, Brain, and Behavior (CMBB), University of Marburg and Justus Liebig University Giessen, Germany; 3 Physics of Cognition Group, Chemnitz University of Technology, Chemnitz, Germany; 4 Cognitive Systems Lab, Chemnitz University of Technology, Chemnitz, Germany

**Keywords:** aging, locomotion, gaze distribution, prior knowledge

## Abstract

Gaze behavior during locomotion must balance the sampling of relevant information and the need for a stable gait. To maintain a safe gait in the light of declining resources, older adults might shift this balance toward the uptake of gait-related information. We investigated how violations of expectations affect gaze behavior and information uptake across age groups by asking younger and older adults to locomote through a virtual hallway, where they encountered expected and unexpected objects. We found that older adults look more on the floor, despite the translational locomotion, though not the rotational, being virtual. Dwell times on unexpected objects were increased in both age groups compared to expected objects. Although older adults showed shorter dwell times on expected objects, dwell times on unexpected objects were similar across age groups. Thus the difference between expected and unexpected objects was greater in older adults. Gaze distributions were more influenced by cognitive control capacities than by motor control capacities. Our findings indicate that unexpected information attracts attention during locomotion—particularly in older adults. However, during actual locomotion in the real world, increased information processing might come at the cost of reduced gait safety if processing resources are shifted away from stabilizing gait.

## Introduction

Avoiding falls is a critical concern, particularly for older adults, as falling can greatly impact health and quality of life. A key factor in preventing falls is the effective use of attentional resources. Whereas the contributions of basic sensory and sensorimotor capacities to fall risks have been documented extensively (e.g., Russell, Taing, & Roy, 2017) and are considered in major prevention programs, the role of attention-related factors to gait safety is less well understood (but see Baek et al., 2021). Gaze direction is a readily accessible proxy for the allocation of attention (Deubel & Schneider, 1996) and in itself subject to age-related changes. We investigate how age modulates gaze when participants negotiate a complex naturalistic environment; specifically, we ask whether and how violations of expectations may draw attentional resources away from information critical for gait safety, such as looks to the floor.

Visual information drives gaze allocation through low-level mechanisms, such as luminance, color, and orientation (Itti, Koch, & Niebur, 1998), as well as higher-level scene properties, such as faces and text (Cerf, Frady, & Koch, 2009) or object properties (Einhäuser, Spain, & Perona, 2008). Several studies have shown that the importance of low-level mechanisms for gaze allocation is attenuated in older adults, with higher-level mechanisms becoming more dominant (Açık, Sarwary, Schultze-Kraft, Onat, & König, 2010; Nuthmann, Schütz, & Einhäuser, 2020). In particular, older adults show an increased reliance on prior knowledge when directing their gaze. When searching for an object, they tend to search longer in locations where the object is expected to be, compared to younger adults (Wynn, Ryan, & Moscovitch, 2020). Similarly, they show a search time benefit when objects are surrounded by associated objects compared to unassociated objects (Ramzaoui, Faure, & Spotorno, 2022). Because these studies have typically considered static scene viewing, it remains an open question how such age-related changes in gaze guidance manifest themselves when observers negotiate a complex naturalistic environment (e.g., during walking).

Previous work has shown that gaze behavior, such as gaze direction, is strongly driven by the task at hand, because individuals prioritize areas that are critical for successful task completion (Buswell, 1935; Einhäuser, Rutishauser, & Koch, 2008; Henderson & Graham, 2008; Rothkopf, Ballard, & Hayhoe, 2007; Yarbus, 1967). Walking introduces specific demands that alter how gaze is allocated (Calow & Lappe, 2008; Einhäuser et al., 2009; Patla, 1997) depending on the difficulty of those demands. For example, gaze behavior is strongly affected by the difficulty of the terrain (Kopiske, Koska, Baumann, Maiwald, & Einhäuser, 2021; Matthis, Yates, & Hayhoe, 2018; 't Hart & Einhäuser, 2012; Thomas, Gardiner, Crompton, & Lawson, 2020) because more challenging surfaces require greater visual attention for safe navigation and the speed of locomotion influences gaze stabilization (Dietrich & Wuehr, 2019). Because the risk of slipping or falling is linked to critical costs in older adults, it can be assumed that gaze allocation should support gait stabilization and safety. Indeed, older adults are found to adopt more cautious visual strategies, for instance, they tend to spend more time looking down at obstacles and initiate anticipatory saccades to future stepping targets earlier than younger adults (Chapman & Hollands, 2006; Chapman & Hollands, 2007; Di Fabio, Zampieri, & Greany, 2003). They also show increased fixation durations on future stepping locations (Zietz & Hollands, 2009) and fixate aspects of the travel path longer than younger adults (Stanley & Hollands, 2014). Paquette and Vallis (2010) found that, when navigating around obstacles, older adults spent more than twice as much time as younger adults looking down at the floor, highlighting this compensatory shift in gaze allocation. Taken together, these findings suggest that older adults spend more resources on monitoring the walking path to compensate for reduced gait stability.

This presumably beneficial, shift in gaze allocation, though, seems to be challenged easily. For example, Zukowski, Iyigün, Giuliani, and Plummer (2020) showed that in busy environments older adults concentrate their gaze on people around them and that this bias is linked to fall risk. Most importantly, the increased reliance on higher-level mechanisms for gaze allocation might critically interfere with safety-oriented gaze behavior. If features or objects in the environment violate prior expectations, older adults might be more biased to focus their gaze on these violations, putting gait stability at risk. In line with predictive processing theory, it is already known that in static scenes, expected objects get identified faster than unexpected ones (Biederman, Mezzanotte, & Rabinowitz, 1982). When trying to memorize a scene, participants fixate unexpected objects earlier, more often and longer than expected ones (Loftus & Mackworth, 1978; Võ & Henderson, 2009). It is also known that younger and older adults have a better memory for unexpected objects than expected ones when instructing them to pay attention to the scene (Mäntylä & Bäckman, 1992; Prull, 2015) and during incidental encoding (Prull, 2015). Not much is known about gaze behavior on unexpected and expected objects in older adults during free viewing. When performing a recognition task where objects were shown in expected or unexpected environments, older adults were significantly slower in identifying if the object belonged to the scene or not, but this effect was amplified during the unexpected trials (Rémy et al., 2013), suggesting that reliance on prior knowledge becomes more pronounced during aging (see Umanath & Marsh, 2014).

Several factors can be considered as contributing to the age-related emphasis on prior knowledge. Given a decline in sensory precision (e.g., Owsley, 2016), a reliance on expectations can be clearly advantageous to information processing. On the other hand, the well-documented age-related decline in cognitive control capacities (West, 1996) might limit the flexible balancing between lower- and higher-level mechanisms. During walking cognitive control contributes to efficiently managing attentional resources. Among other things, individuals must constantly balance their attention between maintaining postural stability, planning safe foot placement, and avoiding obstacles in dynamic environments. The impact of cognitive control resources have been suggested by several studies showing a link between gait stability and cognitive functions (Chapman & Hollands, 2006; Stanley & Hollands, 2014; Uiga, Cheng, Wilson, Masters, & Capio, 2015).

In this study, our main aim is to assess how age modulates the effects of expected and unexpected objects on gaze and incidental memory during locomotion. The experiment was designed using virtual reality to allow for safe locomotion and controlled presentation of unexpected objects. We asked participants to move through a highly realistic virtual hallway, in which expected and unexpected objects were placed, and measured their eye movements. To probe incidental memory, participants were asked after the experiment to recall the objects they had just encountered, a task they had not been made aware of earlier. Additionally, individual cognitive control and motor functions were assessed by standardized tests, given their potential role for strategic gaze allocation. Based on the aforementioned scene-viewing studies, we hypothesized that we would find longer dwell times on unexpected compared to expected objects also during (virtual) locomotion. Under the assumption of predictive processing theory, we furthermore hypothesized that this difference would be enhanced in older adults because of their increased use of world expectations (compared to younger adults), which would potentially lead to longer processing times for unexpected objects. Because of the close connection between attention and memory (Chun & Turk-Browne, 2007; Cowan et al., 2024) we hypothesized that object recall would be enhanced for unexpected objects (given that more attention is allocated to them) and that the difference in recall rate between unexpected and expected objects would be more pronounced in older adults.

## Methods

### Participants

A total of 24 older adults and 24 younger adults (13 women and 11 men in each group) participated in this study. Participants’ ages ranged from 58 to 79 years with a mean of 68.8 years (*SD* = 5.0 years) in the older group and from 20 to 38 years with a mean of 26.1 years (*SD* = 5.3 years) in younger group. Recruitment of participants was managed by calls for participation at the University of Giessen and in local newspapers. All participants had normal or corrected-to-normal vision and no history of ophthalmologic, neurologic, or psychiatric disorders. Older adults were further screened with regard to mild cognitive impairment, using the Montreal Cognitive Assessment Scale. We applied a cutoff score of ≥23, which has been shown to optimize diagnostic accuracy (Carson, Leach, & Murphy, 2018; Elkana, Tal, Oren, Soffer, & Ash, 2020; Nasreddine et al., 2005). None of the older participants had to be excluded based on this criterion. Methods and procedures were approved by the local ethics committee of the Faculty of Behavioral and Social Science, Chemnitz University of Technology, vote V-446-PHKP-WET-"SFB/TRR135.B5"-14052021, and were carried out in accordance with the guidelines of the Declaration of Helsinki (World Medical Association, 2013). Participants provided written informed consent before the experiment and were compensated with course credit or money.

### Assessment of individual differences in cognitive and motor functions

We assessed the cognitive and motor functions of our participants using established measures (Table 1). For the cognitive domain, we focused on cognitive control measures covering the key facets of executive functions (Diamond, 2013; Miyake & Friedman, 2012). These functions include *updating ability*, as measured with the Digit Symbol Substitution Test (Wechsler, 2008), *shifting ability*, as measured with Part B of the Trail Making Test (Kortte, Horner, & Windham, 2002; Reitan & Wolfson, 1986), *inhibition ability*, as measured with the Victoria Stroop Test color naming (Mueller & Piper, 2014; Stroop, 1935), and *nonverbal reasoning ability*, as measured with subtest 3 of the LPS-2 (Kreuzpointner, Lukesch, & Horn, 2013). To obtain a robust, composite measure of cognitive control capacities, we first scaled all the scores so that higher scores correspond to better performance. Then, we *z*-standardized the scores across all participants of both age groups for each task and, by averaging them, calculated an executive function (EF) score for each participant. As indicators for motor functions we measured grip strength and functional mobility, both established measures for frailty (Dudzińska-Griszek, Szuster, & Szewieczek, 2017; Savva et al., 2013; Zhang et al., 2023). Grip strength was measured with a Jamar dynamometer using a standardized protocol (Roberts et al., 2011). Functional mobility was assessed by the Timed Up and Go Test (Christopher, Kraft, Olenick, Kiesling, & Doty, 2021). Finally, we also measured visuomotor coordination and motor speed using the Grooved Pegboard Test (Ruff & Parker, 1993).

**Table 1.** Raw assessment results in older and younger adults,

	Older adults (*n* = 24)		Younger adults (*n* = 24)	
	*M* (*SD*)	*Range*	*M* (*SD*)	*Range*
DSST (raw score)	62.6 (10.8)	41–83	80.7 (11.1)	63–103
TMT-B (s)	75.2 (20.1)	45.7–119.5	44.7 (10.5)	30.5–61.6
VST-C (s)	79.6 (12.1)	55.6–109.2	61.9 (9.2)	49.8–86.8
LPS-3 (raw score)	18.0 (2.6)	15–25	22.5 (2.4)	20–27
GS (kg)	36.0 (10.1)	21–52	41.7 (11.6)	24–62
TUG (s)	5.4 (0.9)	3.7–7.0	5.3 (0.9)	3.6–6.6
GPT (s)	76.3 (11.6)	63.0–101.0	60.1 (6.0)	50.2–73.5

*Note*: DSST = Digit Symbol Substitution Test, WAIS-IV; GPT = Grooved Pegboard Test; GS = grip strength, three attempts, dominant hand, maximum value; LPS-3 = LPS intelligence scale, subtest 3, logical reasoning; M = mean; n = group size; SD = standard deviation; TMT-B = Trail Making Test, part B; TUG = Timed Up and Go Test, functional mobility, rise from an arm chair, walk 3 m, turn, walk back, and sit down again; VST-C = Victoria Stroop Test color naming.

Performance comparisons between older and younger adults using two-sample and two-tailed *t*-tests yielded significant differences on all cognitive measures (i.e., DSST, TMT-B, VST-C, LPS-3), as well as in the visuomotor task (i.e., Grooved Pegboard Test [GPT]), all *p*s < 0.001. In contrast, measures targeting frailty (i.e., grip strength [GS] and Timed Up and Go Test [TUG]) showed no age-related differences, with *p* = 0.080 and *p* = 0.785, respectively.

### Equipment and VR model

We used the HTC Vive Pro Eye Headset to present a VR environment. This head-mounted display with built-in eye tracking has been shown to be well tailored for research and to provide high-quality gaze data, even when participants wear individual visual corrections (i.e., contact lenses or glasses) (Hou, Abdrabou, Weidner, & Gellersen, 2024; Schuetz & Fiehler, 2022). The headset has the following technical specifications (HTC Corporation, 2024, September 25): The display has a resolution of 1440 × 1600 pixels per eye, a refresh rate of 90 Hz, and a field of view of 110°. The integrated eye tracker runs at a sampling rate of 120 Hz and achieves a spatial accuracy of 0.5° to 1.1°. We used the eye tracker's automated five-point calibration protocol and implemented an additional five-point validation at the end of each run (see section Procedure).

The VR environment was implemented using the software packages Blender (version 3.1) and Unity (version 2019.4.32f1). The SteamVR plugin (version 2.7.3) was used as interface with Unity. The SRanipal software development kit and Runtime (version 1.3.2.0) provided access to the eye tracker data. For data collection, we used a laptop that was configured to meet our specific requirements (XMG NEO 17, AMD Ryzen 9 5900HX CPU, 64 GB RAM, NVIDIA GeForce RTX 3080 GPU). Overall, four SteamVR 2.0 base stations (i.e., lighthouses) were set up close to the ceiling, one in each of the four corners. The lighthouses allowed for tracking the position of the display and the movement of our participants throughout the experiment.

Virtual movement was implemented using an established method that has been shown to allow naturalistic movement in virtual reality (Feder, Bendixen, & Einhäuser, 2022). For rotational movement, the method uses the actual rotation of the participants’ head and body to allow them to turn and look around while in VR while translation through the environment is simulated and controlled with a button press. The participants were equipped with a Valve Index Controller and a Vive Tracker 3.0. They held the Valve Index Controller in their right hand and pressed the trigger button with their index finger to simulate forward movement. The physical rotation of the participants’ body was tracked via the Vive Tracker 3.0 that was placed on the right hip of the participants. Additionally, we implemented two measures to minimize the risk of VR induced motion sickness. First, the movement speed was locked at 0.6 m/s for both age groups. In addition, we implemented a damped acceleration and deceleration, respectively, during the start and stop of the movement.

**Figure 1.**
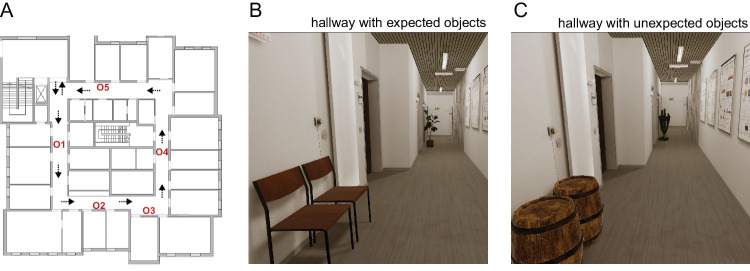
(**A**) Floor plan and object positions (O1 to O5); small arrows indicate the instructed locomotion route. (**B**) and (**C**) Illustration of the same virtual hallway section with panel (**B**) showing the expected objects (chairs and plant), and panel (**C**) showing the matching unexpected objects (barrels and cactus); viewing angle is chosen to show two example objects at once and is not representative for viewing angles in the experiment.

The VR environment was modeled in detail based on an actual hallway in the psychology department at the University of Giessen, which is representative of a typical university or office building hallway. Along the hallway, objects were placed at five different positions (Figure 1A).

**Figure 2.**
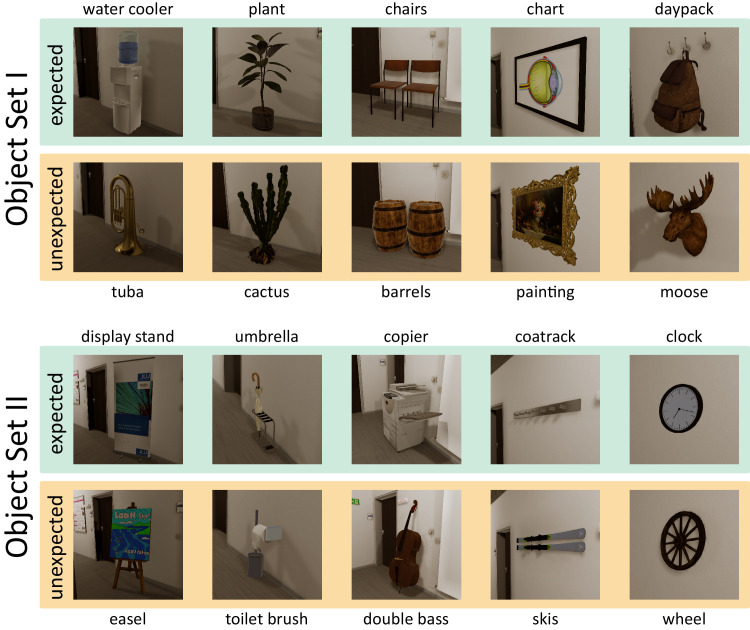
Overview of object pairs. Overall, there are four different hallway layouts, of which two are matched with each other in terms of the objects’ shape, size, and color, resulting in object set I and object set II. Expected layouts are shown on a green background, unexpected layouts are shown on an orange background. Objects were placed at the indicated locations, with the leftmost column corresponding to O1 in Fig. 1A, and the rightmost to O5.

We rendered 10 object pairs in which one object was expected within the context of a university hallway, and the other one was unexpected. Since many factors beyond this *expectedness* manipulation can be expected to affect gaze, we chose the objects such that within each pair the expected and unexpected object were as closely matched as possible in terms of their appearance (size, shape, color). In contrast, we aimed for a high variability between the different object pairs in such potentially gaze-driving visual attributes, without violating naturalness and the (im)plausibility of the (un)expected objects in the context. With our experimental design and a priori analysis (see section Procedure), we ensured that exactly those objects were included in the analysis for which a given observer had encountered both objects of the pair. Specifically, we used the pairs to create four different hallway layouts: two layouts each containing five expected objects, and two layouts each containing the corresponding five unexpected objects. For each pair, the expected and unexpected objects were placed at identical locations (O1 to O5 as seen in Figure 1A) across the layouts. Figure 2 provides an overview of all object pairs, shown in their hallway context as used in the experiment. Figures 1B,  1C give an example of two matched hallway sections, each showing two object pairs at their designated locations.

### Procedure

All participants were familiarized with the VR equipment, and we provided a detailed introduction to the technical procedures. We matched the participant's height in the virtual environment with their actual height by measuring eye level and specifying this value in the VR model. Before data collection, participants completed a practice run in a section of the hallway that was not part of the experiment and contained no objects. The practice run allowed the participant to practice controlling the virtual movement and helped us to ensure that all participants felt comfortable in the virtual environment.

**Figure 3.**
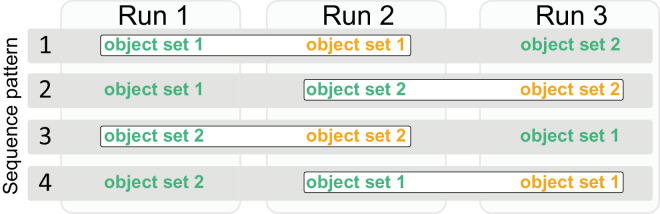
Overview of the four possible sequence patterns of hallway runs; each sequence was used for six participants per age group. Expected object sets are given in green, unexpected objects sets are given in orange. The pairs outlined in white boxes show the matched expected and unexpected layouts which were used in the analysis (see section Data analyses for details).

Participants were instructed to move through the hallway following a counterclockwise route, as depicted in Figure 1A. They were asked to locomote as continuously as possible without stopping. Locomotion was performed without an additional task; the presence of objects was not mentioned in the instruction. All participants completed three runs through the hallway. The three runs included a sequence of expected and unexpected layouts (Figure 3). The sequence always comprised of the matched expected and unexpected layouts from one object set plus the expected layout from the other object set (compare Figure 2). The order of the matched layouts was fixed so that the expected layout always preceded the unexpected layout. The matched layouts could be either preceded or followed by the expected layout of the other object set. This procedure was chosen to enable a direct comparison of gaze behavior in matched expected and unexpected layout for each participant. At the same time, we aimed to avoid putatively critical order effects. We reasoned that surprise effects in unexpected layouts could carry over to the following expected layout (i.e., attention could be drawn to locations where unexpected objects have been encountered before). Thus we decided to use a fixed order in which the expected layout always preceded the unexpected layout. We further aimed to address practice effects. To avoid that the expected comparison layout was consistently the initial run, we included sequences in which the matched layout was preceded by the expected layout from the other object set, respectively. Overall, this procedure resulted in four possible sequence patterns (Figure 3) that were balanced across all participants within each age group. Before and after each run, a calibration was performed to ensure the reliability (accuracy) of gaze data.

After completing the sequence of three runs and removing the VR equipment, participants were asked to recall as many objects as possible that they had encountered in the virtual environment. Importantly, our instructions did not mention that the participants had to attend to the objects in the environment nor did it mention that the participants would be asked to recall them.

### Data analyses

We defined the objects and the ground floor in our virtual environment as relevant areas of interest for our analysis of gaze allocation during locomotion. We calculated overall dwell time on objects and on the ground floor for the matched expected and unexpected runs. The additional expected run from the respective other object set within each sequence was not analyzed (compare Figure 3).

Dwell time on the objects and the ground floor, as well as the trial durations, were analyzed using mixed analyses of variance (ANOVAs) with the between-subject factor *age group* (levels: older, younger) and the within-subject factor *expectancy* (levels: expected, unexpected). Because of differences in overall trial duration between age groups (see Results: Trial duration and movement trajectories), we based all quantitative analyses on normalized dwell times, which we defined as the aggregate dwell time on the objects in the trial divided by the total trial duration (object dwell time) and the dwell time on the floor divided by the total trial duration (floor dwell time). The procedure controls for potential confounds that could emerge from absolute duration differences. In addition, we calculated the ratio of object dwell time to floor dwell time, providing a measure for balancing gaze allocation between both areas of interest. This “object-to-floor dwell time ratio” is insensitive to potential effects of different VR-navigation strategies, as these would likely affect floor dwell time and object dwell time to the same extent. Normalized dwell times and the object-to-floor dwell time ratio were arcsine-square-root transformed for variance stabilization and were used in the mixed ANOVAs described above. The relationship between the object-to-floor dwell time ratio and the measured executive functions, as well as the measured motor parameters, were investigated by correlational analyses. Finally, we analyzed the participants’ performance on the object recall task performed at the end of the experiment using a mixed ANOVA with the factors *age group* and *expectancy*. We report uncorrected *p*-values, and generalized eta-squared as measure of effect size, for which 0.02 is conventionally considered a small effect, 0.13 a medium effect and 0.26 a large effect (Bakeman, 2005). A significance level of α = 0.05 was applied for all statistical analyses, and tests were two-tailed. For post-hoc comparisons, uncorrected p-values were reported, but significance was evaluated based on Bonferroni-Holm corrected *p*-values. Data analyses were carried out with Matlab (R2022b), as well as RStudio (2023.06.1).

## Results

We investigated the dwell times on matched expected and unexpected objects in older and younger adults. Age effects on gaze allocation were then analyzed in detail for the expected and unexpected runs. Differences in gaze allocation were scrutinized considering the role of executive functions and motor functions.

### Overview of dwell times for object pairs

**Figure 4.**
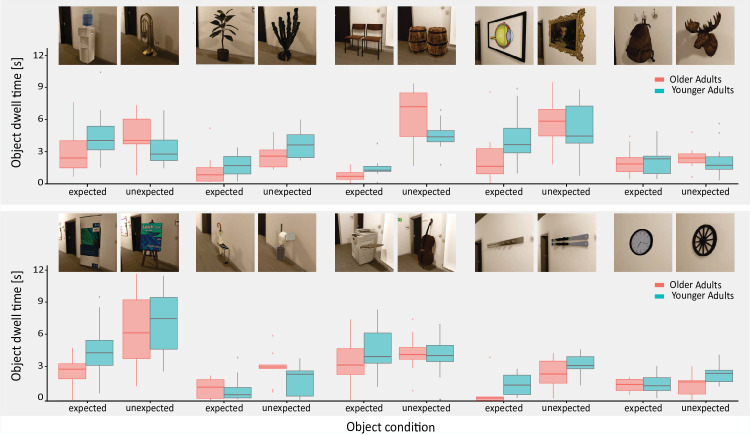
Total absolute (unnormalized) dwell time per object in seconds of all participants grouped by object pairs and by age group, respectively. Shown are boxplots that include the median, as well as the 25% and 75% quartiles. Whiskers extend to the smallest/largest value no further than 1.5 times the interquartile range from the lower/higher quartile. Data outside of this range are plotted as outliers.

Both objects of each object pair were seen by 12 participants per age group (Figure 3). A first inspection of the data per object and age group shows a general pattern of shorter dwell times on objects for the older adults compared to the younger adults, as well as longer dwell times on unexpected objects than on expected objects (Figure 4). This holds despite some substantial variability between different object pairs. Notably, this variability is generally larger than that observed between the objects of the same pair, reassuring that the visual matching was successful.

In summary, this initial qualitative inspection of dwell time patterns suggests that, despite variability across our object pairs, our choice of objects in each object set consistently induced an overall shift in gaze allocation. In both age groups the chosen unexpected objects attracted gaze, indicating a violation of participants’ expectations.

### Trial duration and movement trajectories

**Figure 5.**
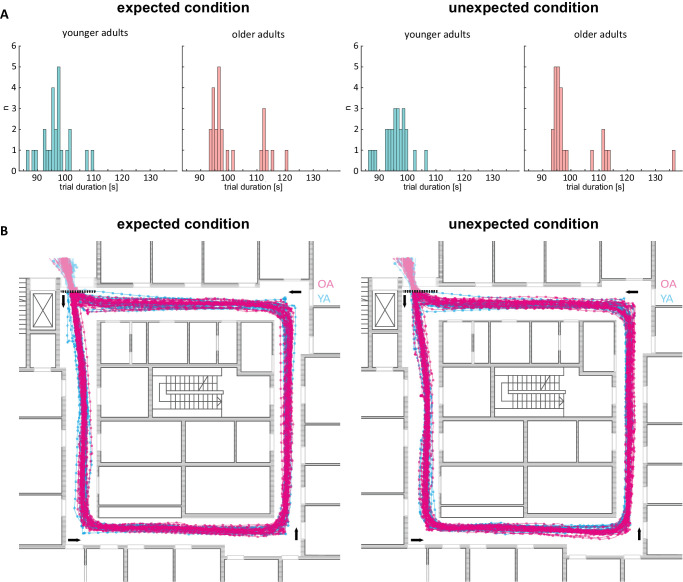
(**A**) Distribution of trial duration in seconds for younger and older adults for the expected and unexpected condition. (**B**) Participant's trajectories: projection of the participants’ head positions on the ground floor; sampling rate of 120 Hz, dots mark 1-s intervals. Participants enter and exit the hallway in the top left corner and move counterclockwise; trial duration starts and ends when crossing the coordinate depicted as dashed line from “above” and “below”, respectively. OA = older adults; YA = younger adults.

To ensure that any differences in how participants negotiated the VR environment did not affect our gaze results, we measured trial durations and assessed the participants’ movement trajectories through the environment. Trial durations were rather variable (Figure 5A). On average, older adults took longer to negotiate the VR environment (expected condition: M = 96.6 s, *SD* = 5.3 s in younger adults vs. M = 101.4 s, *SD* = 8.7 s in older adults; unexpected condition: M = 95.6 s, *SD* = 4.5 s in younger adults vs. M = 100.6 s, *SD* = 10.2 s in older adults). A two-factor ANOVA with *age group* as a between-subject factor and *expectancy* as a within subjects repeated factor*,* yielded a significant main effect of *age group* (*F*(1, 46) = 5.55, *p* = 0.02, η² = 0.10), which supports the use of normalized dwell times for our main analyses to minimize potential confounds. Importantly, we did not observe a main effect of *expectancy* (*F*(1, 46) = 1.43, *p* = 0.24, η² = 0.004) nor an interaction between the two factors (*F*(1, 46) < 0.001, *p* = 0.99, η² < 0.001). This ensures that any effects of *expectancy* on dwell times cannot be confounded by systematic differences in negotiating the VR environment.

As the translational speed in the VR environment was fixed, differences in trial duration could in principle result from different trajectories or from occasional stopping. In most cases stopping was brief, in 37 of 96 total trials no stopping occurred, in 69 of 96 trials stopping lasted less than 1 s. Visual inspection of all first-person replays and trajectories showed that stopping almost exclusively occurred at bends (the corners of the corridors) and never when approaching an object. In line with this observation, inspection of the shape of the trajectories does not suggest a qualitative dependence on *age group* or *expectancy* (Figure 5B).

### Effects of age group and object expectancy on gaze allocation

**Figure 6.**
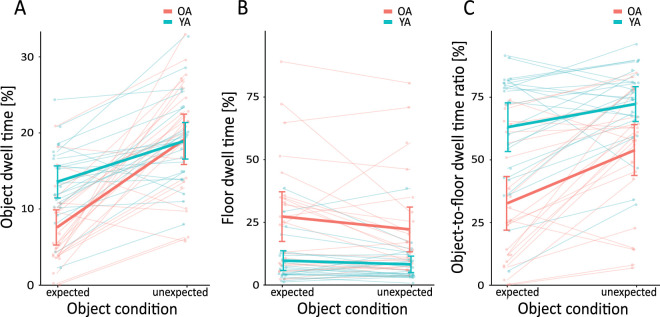
(**A**) Percentage of time spent looking at objects (normalized object dwell time) as a function of age group and expectancy of objects. (**B**) Percentage of time spent looking on the ground floor (normalized floor dwell time) as a function of age group and expectancy of objects. Note the different scale of the y-axis compared to panel A. (**C**) Object-to-floor dwell time ratio as a function of age group and expectancy of objects. Transparent dots and lines show individual participant data. Bold lines show the mean for each age group. 95 % confidence intervals are plotted. OA = older adults; YA = younger adults.

Next, we analyzed quantitatively whether normalized object dwell times during locomotion were affected by the age of our participants and the expectedness of the objects (Figure 6A). We ran a two-factor ANOVA with *age group* as between-subject factor and repeated measures on the factor *expectancy*. The main effects for *age group*, *F*(1, 46) = 6.36, *p* = 0.015, η^2^ = 0.09, and *expectancy*, *F*(1, 46) = 72.81, *p* < 0.001, η*^2^* = 0.33, reached significance, but were qualified by a significant interaction, *F*(1, 46) = 12.80, *p* < 0.001, η^2^ = 0.08. Follow-up *t*-tests showed that dwell times on unexpected objects were longer than on expected objects both in older and younger adults (*t*(23) = −6.83, *p* < 0.001, *d* = 1.68, and *t*(23) = −5.35, *p* < 0.001, *d* = 0.99, respectively). Notably, the longer dwell times on unexpected objects was more pronounced in older adults: Although dwell times on expected objects were significantly lower in older compared to younger adults, *t*(46) = 3.95, *p* < 0.001, *d* = 1.14, dwell times on unexpected objects were comparable between both age groups, *t*(46) = 0.10, *p* = 0.924, *d* = 0.03.

Dwell times on the ground floor as a function of *age group* and *expectancy* are given in Figure 6B. The mixed ANOVA yielded a main effect of *age group*, *F*(1, 46) = 13.43, *p* < 0.001, η*^2^* = 0.21, indicating longer dwell times on the floor in older adults, and a main effect of *expectancy*, *F*(1, 46) = 4.08, *p* = 0.049, η*^2^* = 0.009, indicating shorter dwell times on the floor when objects were unexpected. There was no interaction between both factors, *F*(1, 46) = 0.86, *p* = 0.356, η^2^ = 0.002.

In order to analyze the balance between gaze allocation to objects and the ground floor, we further analyzed the ratio of dwell times on each area of interest (i.e., the objects and the ground floor). Figure 6C illustrates this relative measure (object-to-floor dwell time ratio) as a function of age group and expectancy of objects. A mixed ANOVA yielded a significant main effect of *age group*, *F*(1, 46) = 17.81, *p* < 0.001, η^2^ = 0.24, and *expectancy*, *F*(1, 46) = 29.05, *p* < 0.001, η^2^ = 0.11. However, both main effects were qualified by a significant interaction, *F*(1, 46) = 4.95, *p* = 0.031, η^2^ = 0.02. Follow-up *t*-tests indicated that younger adults consistently balanced their gaze more towards objects than older adults (expected condition: *t*(46) = 4.33, *p* < 0.001, *d* = 1.25; unexpected condition: *t*(46) = 3.16, *p* = 0.003, *d* = 0.91). In both age groups the proportion with which gaze was allocated to objects compared to the floor increased in the unexpected condition (older adults: *t*(23) = −4.80, *p* < 0.001, *d* = 0.87; younger adults: *t*(23) = −2.60, *p* = 0.016, *d* = 0.47), but this effect is less pronounced in younger adults.

### Relationship between cognitive control capacities, as well as motor function with gaze allocation

**Figure 7.**
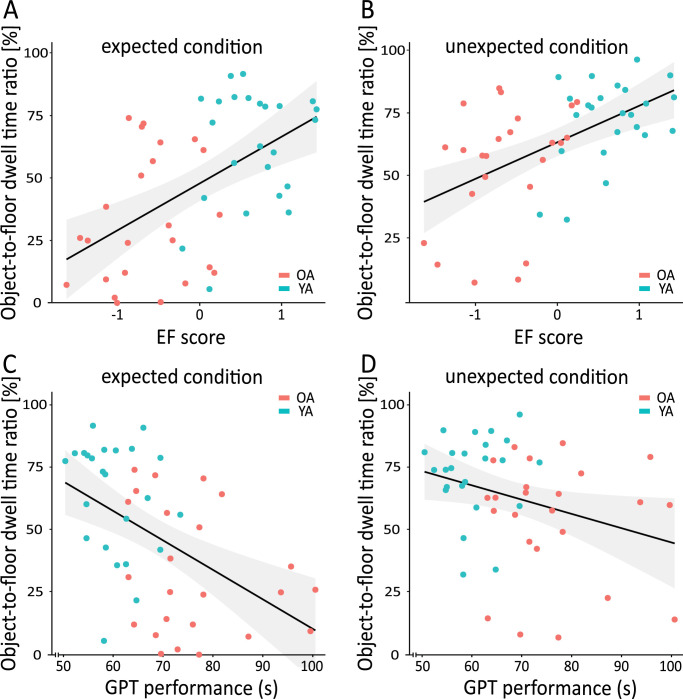
Relationship between the object-to-floor ratio and the EF score in the expected condition (**A**), the unexpected condition (**B**), as well as the performance during the GPT in the expected (**C**) and unexpected condition (**D**) for both age groups. The bold black line shows the best linear fit, and the gray area around it shows the 95% confidence interval. OA = older adults; YA = younger adults.

Next, we analyzed the role of cognitive control capacities as well as motor functions on gaze allocation during locomotion. Cognitive control capacities were captured by an executive function (EF) score covering key facets. Figure 7A and 7B illustrate the link between the object-to-floor dwell time ratio and the EF score for the expected and unexpected conditions, respectively. We found significant correlations for both conditions (expected condition: *r*(46) = 0.54, *p* < 0.001; unexpected condition: *r*(46) = 0.53, *p* < 0.001). EF scores explained more than 30% of the variance in this relative measure. When regarding Figure 7, differences in age group membership suggest that this correlation is not merely driven by group differences but actually describes a general link. A partial correlation analysis controlling for the factor *age group*, though attenuating the correlation, showed for the unexpected condition the correlation between EF and object-to-floor dwell time ratio is robust, *r*(45) = 0.36, *p* = 0.014. For the expected condition, the correlation fails to reach significance when controlling for age group, *r*(45) = 0.21, *p* = 0.160.

Correlational analyses considering assessed motor functions indicated only minor associations with gaze allocation. Neither grip strength (GS) nor function mobility (TUG) correlated with relative gaze allocation in any condition (all *p*s > 0.440). For visuomotor coordination and motor speed (GPT), we found a significant correlation in the expected condition (*r*(46) = −0.48, p < 0.001) as well as in the unexpected condition (*r*(46) = −0.31, p = 0.035) (Figure 7C, 7D). However, when controlling for *age group*, both correlations fail to reach significance (both *p*s > 0.353). In summary, our findings suggest that age-related differences in gaze allocation are driven by cognitive control capacities, but only to a minor degree by individual differences in motor functions.

### Object memory

**Figure 8.**
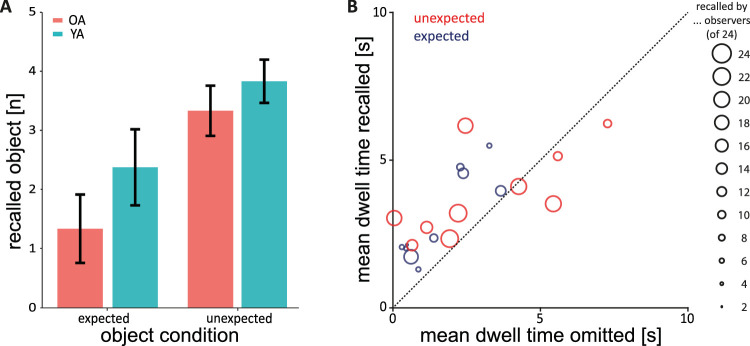
(**A**) Number of recalled objects as a function of age group and expectancy condition (i.e., expected and unexpected objects). 95% confidence intervals are plotted. OA = older adults; YA = younger adults. (**B**) Dwell time on omitted objects plotted against dwell time on recalled objects. Circle size indicates how many participants recalled the object. Unexpected objects got recalled by more participants.

We analyzed how free recall of encountered objects during the unannounced recall task varied across age groups and expectancy of objects. The number of recalled items were submitted to a mixed ANOVA with the between-subject factor *age group* and the within-subject factor *expectancy* (Figure 8A). We found a main effect of *age group*, indicating an age-related memory performance decrease, *F*(1, 46) = 6.72, *p* = 0.013, η^2^ = 0.09. The main effect of *expectancy* was also significant, showing better memory performance for unexpected objects, *F*(1, 46) = 82.57, *p* < 0.001, η^2^ = 0.34. There was no interaction between the factors, *F*(1, 46) = 2.03, *p* = 0.161, η^2^ = 0.01. For the majority (16/20) of objects, the mean dwell time across observers for objects that were recalled was larger than the mean dwell time across observers who omitted (i.e., forgot) the object (Figure 8B), and this difference was significant (*t*(19) = 3.66, *p* = 0.002), suggesting that observers were more likely to recall an object when they had dwelled on it longer.

## Discussion

In this study, we investigated how age influences gaze guidance and whether younger and older adults differ in their allocation of attention to unexpected objects in their environment. Additionally, we examined how cognitive and motor capabilities contribute to gaze allocation as well as how object recall is influenced by age and the expectedness of objects in the environment. Previous research has explored the role of object expectancy in visual attention using line drawings (Biederman et al., 1982; Henderson, Weeks, & Hollingworth, 1999; Loftus & Mackworth, 1978) and photos (Võ & Henderson, 2009). Building on this research, we aimed to achieve higher ecological validity by using a highly realistic virtual environment to investigate gaze guidance in a complex, dynamic setting.

Virtual reality allows us to design detailed realistic environments while still maintaining control over the stimuli and their presentation. Using a highly realistic model is necessary to achieve the effect of unexpected objects being unexpected. However, it is important to acknowledge that virtual reality cannot fully replicate the real world (Kim & Rhiu, 2021; Savino et al., 2019). In general, the ability to move freely enhances immersion in a virtual environment. However, the size of the virtual environment in our paradigm did not allow unrestricted movement in the actual world while being immersed in VR. As a compromise, we used a hybrid method that combined simulated translational movement with real rotational movement (Feder et al., 2022), tracking body position and rotation on the hips. This approach has been shown to yield more consistent path trajectories compared to choosing direction with the head or joystick; and to provide a safe, and controllable proxy for the real world (Drewes, Feder, & Einhäuser, 2021). Although participants were not able to walk in place to locomote, selecting the direction of locomotion by physically rotating the body remained intuitive. The VR headset's field of view and resolution were sufficient to present the hallway and objects clearly, as supported by participants’ successful object recall (Figure 8). Combined with the movement control practice, participants locomoted through the virtual environment in a controlled manner.

Our results revealed several key patterns: Older adults spent significantly more time looking at the floor than younger adults, regardless of the environment's expectedness (i.e., whether the corridor contained expected or unexpected objects). However, both age groups reduced their floor-gaze when objects in the environment were unexpected. Both younger and older adults allocated more visual attention to unexpected objects than to expected ones, with older adults showing a pronounced increase in dwell time for unexpected objects. Executive function was positively correlated with gaze allocation to objects in both expected and unexpected environments. Fine motor function showed a negative correlation with gaze allocation in these contexts, though overall motor function showed only minor associations. Finally, although younger adults outperformed older adults in object recall, both groups showed better recall for unexpected objects.

Our results showed that older adults spent significantly more time looking on the floor compared to younger adults, regardless of the environment's expectedness. This suggests that age-related differences in gaze allocation may be driven by heightened focus on walking safety, which is in line with findings about gaze behavior in older adults during locomotion (for review see Uiga et al., 2015). Notably, this behavior occurred even though participants were navigating a virtual environment without real objects or challenging terrain. This raises the question whether floor-gazing is a habitual behavior learned from navigating real-world environments, where safety concerns are more pronounced (Matthis et al., 2018; 't Hart & Einhäuser, 2012). Impaired visual acuity (Hallemans, Ortibus, Meire, & Aerts, 2010), proprioception (Ghez & Sainburg, 1995) and vestibular function (Anson, Pineault, Bair, Studenski, & Agrawal, 2019) and the slowing of reaction time (Hardwick, Forrence, Costello, Zackowski, & Haith, 2022) are some of the natural changes that lead to an unstable gait in the real world, which can have an impact on gaze behavior in virtual reality. In the present study, participants experienced real vestibular rotational input but no vestibular input related to forward movement because forward movement was simulated via visual presentation with the virtual reality headset. This discrepancy between vestibular and visual input may have caused participants to adopt a less stable stance compared to actual walking, increasing floor-gaze compared to real-world settings. This, however, would affect both age groups.

This safety-oriented gaze pattern may also explain age related differences in how older adults process expected and unexpected objects. In a familiar environment when expected objects are presented, we observe that older adults have a lower object dwell time than younger adults. This is in line with the previously stated finding that older adults look more on the ground and therefore explore the environment less during locomotion. When presented with unexpected objects, we find an increase in object dwell time in both age groups but a steeper increase in older adults. The general increase in dwell time can be explained by the novelty (with respect to the present context) of the objects that attracts attention in younger and in older adults alike. The steeper increase in attention in older adults might be explained with predictive processing theory. Although both age groups experience prediction errors and therefore take longer to interpret the percept, older adults have stronger priors than younger adults. This increased reliance on priors and expectations might therefore lead to older adults being more affected by predictive errors. Additionally, reduced processing speed, which is part of naturally occurring cognitive decline (Sánchez-Izquierdo & Fernández-Ballesteros, 2021), increases the time needed to process unexpected information and might amplify the steeper increase in dwell time on unexpected objects in the older adults.

To mimic the diversity of naturally occurring objects, we aimed for a high degree of variability between our objects in terms of their visual appearance (e.g., size, color, shape). Importantly, however, we ensured that objects *within the same pair* were matched as closely as possible in terms of their visual attributes. By our experimental design and analysis, we ensured that either both or none of the two objects in a pair entered the analysis for a given participant, thereby mitigating any effects of potential differences induced by the necessarily small number of object pairs in combination with the high diversity between pairs. The fact that object dwell times were typically more variable between object pairs than within a pair (cf., Figure 4) confirms the success of this manipulation. Although it cannot be ruled out that the objects within a pair differed in other relevant attributes for gaze (although the matched size, location and low-level salience are among the most critical ones, Stoll, Thrun, Nuthmann, & Einhäuser, 2015), because the viewed objects were identical across the age groups, such factors are unlikely to confound the *age group* effects and their interaction with *expectancy.* The design also necessitated that analyzed expected objects were presented before the unexpected objects, though we were able to mitigate some of the potential order effects with the “filler” block (the other set of expected objects, cf., Figure 3), and the balancing across groups again renders any confounds on *age group* effects and interactions unlikely.

With respect to age effects, we consider the “surprise” value of an unexpected object to play a similar role in our task as visual (physical) salience does in scene viewing. Our results are consistent with the earlier observation that the preference for higher-salience objects over lower-salience objects is increased in older compared to younger adults (Nuthmann et al., 2020), despite a general lowering of salience effects with age (Açık et al., 2010). In the current experiment, higher salience through unexpectancy has a larger effect on gaze in older than in younger adults, similar to higher visual salience having a larger effect on gaze-based object selection in scene viewing. Our data also provide an alternative explanation to these earlier findings: it is not that older adults are attracted by saliency less, but rather that their processing is bound by other constraints (such as safe gait), despite those being irrelevant in scene viewing with fixed head and body, which keeps them less attracted to salient scene regions, while still using salience for object prioritization.

Hand grip strength and performance on the TUG test did not show any relationship with the gaze distribution—better motor capabilities did not lead to more dwell time on the objects. This goes against the idea that better motor function would lead to more available resources for exploring the environment and therefore increasing the gaze distribution on the objects. Especially because hand grip strength is a useful biomarker for overall fitness and strength and there are established links between reduced hand grip strength and a limited ability during walking, climbing stairs and standing up from chairs (Forrest, Williams, Leeds, Robare, & Bechard, 2018) as well as falls (Yang et al., 2018). The performance during the TUG has been found to be linked to fall risk (Viccaro, Perera, & Studenski, 2011). However, systematic reviews question the informative value of the TUG regarding the prediction of the risk of falls in older adults (Barry, Galvin, Keogh, Horgan, & Fahey, 2014; Beauchet et al., 2011), especially in healthy older adults (Schoene et al., 2013). The fact that our sample is restricted to healthy adults could therefore explain why we did not find any correlation between the motor abilities and gaze distribution in our participants. We were specifically interested in healthy aging and made sure all our participants performed above commonly used cutoff scores during all cognitive, motor, and sensory tests during the initial assessment.

We did, however, find a relationship between the performance on the GPT and gaze distribution. The GPT is commonly used to measure motor function, specifically fine motor skills. Better performance during the test correlates with longer dwell time on the objects, indicating more leftover resources for eye movements for exploration. The fact that the correlation between GPT and gaze distribution was not age independent suggests that the relationship might not be a direct one. Although the GPT is a commonly used as a measure for fine motor skills, it can also be used as an indicator for cognitive control capacities (Tolle, Rahman-Filipiak, Hale, Kitchen Andren, & Spencer, 2020). Completing the test involves visual-spatial skills, executive functions like planning and decision-making, as well as constant concentration.

Thus, it is not surprising that we find a relationship between the executive function score of our participants and their gaze distribution—even when controlling for age group. The higher the EF score, the longer the time spent looking at objects, implying that more executive functioning leaves more resources to perform exploratory eye movements during locomotion. When navigating complex dynamic environments, higher-order neurological processes are active, to divide attention and plan movements, making walking a complex task (Hausdorff, Yogev, Springer, Simon, & Giladi, 2005). The cognitive demands of completing motor tasks increase during aging, but at the same time cognitive resources decline (Seidler et al., 2010). A relationship between cognitive impairment, specifically executive function, and increased fall risk has been shown (Muir, Gopaul, & Montero Odasso, 2012). Our findings emphasize that gaze distribution is influenced by cognitive control capacities rather than motor control capacities.

Younger adults, who viewed objects generally longer than older adults, show a better ability to recall the objects. Similarly, unexpected objects, which were viewed longer than expected objects, were also recalled better. Memory performance is likely influenced by viewing duration. The memory advantage for unexpected objects aligns with previous studies (Hess & Slaughter, 1990; Mäntylä & Bäckman, 1992; Prull, 2015; Wynn et al., 2020) when no explicit instructions are given about how expected objects would be in the context. This reinforces the idea that unexpected objects naturally capture attention, leading to better encoding.

## Conclusion

We found further evidence that for gaze selection older adults place more value on maintaining a stable gait under normal circumstances. When confronted with an unexpected environment they shift this priority to exploring their environment to a degree similar to younger adults. The larger increase in dwell time for unexpected objects among older adults suggests they may struggle more with filtering out irrelevant information, potentially affecting their ability to navigate the real world effectively. This might reduce gait safety, making it more difficult for older adults to maintain their balance and avoid obstacles. Consequently, this can pose a disadvantage for older adults, potentially increasing the likelihood of falls and other navigation-related accidents that do not only have physical but also psychological and social consequences (Petersen, König, & Hajek, 2020). Our findings highlight the need to consider environmental expectedness in designing spaces for older adults, as unexpected features may disrupt attentional allocation patterns.
